# How Can the Spread of Scarlet Fever and Diphtheria Be Prevented in Public Schools?

**Published:** 1899-07

**Authors:** Gilman Robinson

**Affiliations:** Atlanta, Ga.


### HOW CAN THE SPREAD OF SCARLET FEVER AND DIPHTHERIA BE PREVENTED IN PUBLIC SCHOOLS.*

BY GILMAN ROBINSON, M.D.,
ATLANTA, GA.

When our president telephoned me the other day and asked me to read a paper on this subject, it seemed to me for a moment that I could not have time to write anything of value. On consideration, however, I concluded that even if what I might say were not new, yet kindly criticism from my point of view would at least bring out a good and full discussion, and that we might get out some suggestions that would be of value to the Board of Health and hence to the city of Atlanta. If at any time in this paper I may criticize some of our methods, it is done simply to bring out discussion, and not in any spirit of mean criticism. After receiving my summons from our president I wrote at once to Boston, on whose board I have some friends, asking for the methods of protection in that city. The protection given there I will speak of later on.

The influence of schools upon the spread of infection, and the need of public care over the schools, has been much discussed in all large centers, and with a general agreement that large bodies of people collected in small space conduce to the spread of infectious diseases. This is particularly true of our school children, whose age and familiar habits render them particularly liable to the infectious diseases as a prolific nucleus for the spread and extension of these diseases. We all agree, I am sure, that the infective agent is often present in our schools, and it only remains to show how the infection is caused. For our purpose this evening diphtheria and scarlet fever will be sufficient for us to consider. Before going on further with our discussion let us consider for a moment the different methods of infection of some of the more common contagious diseases. It was thought from the beginning of our

*Read before the Atlanta Society of Medicine. May 18, 1899.

knowledge of diphtheria until within a few years, that it belonged to the so-called filth diseases, and with this end in view many boards of health have carried out different lines of work trying to prove or disprove the theory of unsanitary conditions as the cause. For the last fifteen years the boards of health of our great cities have been carefully trying to find any relation between street gullies, perforated sewer covers, sewer outfalls, low, damp ground, proximity to tide-water, flats poorly constructed and badly-kept houses, filth and defective drainage, as the source of diphtheritic infection, but with absolutely negative results. Hence their conclusion that diphtheria is communicated directly from person to person, or indirectly by some intermediate article upon which the infective matter may have been lodged, and where it may remain active for a greater or less time. Dr. Burgin, chairman of the Board of Health of Boston for many years, has shown conclusively that much of the infection of diphtheria takes place in the public schools, and of the kindergarten schools he says: "The danger of spreading the disease by a single case is much greater, because thus children, by virtue of the different processes of teaching, are brought into much closer relations to each other, and they use a larger number of objects in common which are very liable to become infected." One unrecognized case under such circumstances may give rise to a dozen more, and without ever being able to trace one of them to its particular source. So much for diphtheria; now briefly as to scarlet fever and measles. Measles is a disease particularly contagious in its very early stages. Its greatest virulence is in its very beginning, contagion even taking place before the rash appears. Scarlet fever, on the other hand, is not particularly contagious in its early stages, even when the rash is at its height. But as the child gets well and the desquamation begins, then does it become a very hot-bed of contagion; then little minute particles of skin are too easily carried in the clothes, hair, or scattered to the four winds by the shaking of some article from the window, which I feel sure has been done in this city in at least one instance. Apropos of the contagiousness of the two diseases, let me relate the case of two children, a little boy and girl. The little boy was taken with scarlet fever and the little girl, who had slept with him for two nights, until the diagnosis was positively made, was sent off to the country, where she did well, until a month later, her little brother nearly well, wrote her a letter, which was allowed to remain under her pillow. Four days later she came down with scarlet fever. The following year the little boy was taken with measles, and his sister was again sent away. But in a few days she came down with the disease. These two cases illustrate the difference in contagion of the two troubles.

Having now considered the liability of contagion, and having shown the ease with which it may be communicated, let us first consider what precautions are taken by some of our larger cities. I have taken the city of Boston as an example particularly because I have friends on its board who would give me much desired information, but chiefly as it was the first city to adopt the daily inspection of schools by physicians, and whose laws and regulations have been practically adopted in the whole or in some modified form by the other large cities. This daily inspection has been in force for some three years, and its results have been largely in favor of its permanent continuance. One other significant fact that their statistics show is, that the number of contagious diseases reported during the summer months of vacation in the schools is much smaller. For instance, for nineteen years the cases of diphtheria reported for each of the school months was 2,772, and for each of the four months of little or no school, only 2,077. For scarlet fever the difference is even greater, 2,779 against 1,191 in vacation. A fact I do not, however, attribute entirely to the closing of the schools, but it is, I believe, an important factor. I do not think that I can better tell you of the methods used in Boston than by briefly giving you the gist of a circular sent out by the board of health to the physicians of Boston.

"*To the Physicians of Boston:*

"GENTLEMEN:—The Board of Health respectfully calls your attention to the enclosed regulations and cards which have been prepared, and are being used, with other means, to check the spread of contagious diseases.

"The Board of Health has the authority and is charged with the duty, under the statute law, of taking official charge of any case of contagious or infectious disease which may be dangerous to the public health. It prefers that such cases only as cannot be properly isolated at home be sent to the hospital, and that all others be attended by the family physician at home, and nursed and provided for by any safe and proper method which may be chosen by the family. The isolation of such cases at home must be satisfactory to the Board of Health, and be so certified by its authorized medical agent in the district in which patient is found. Such patient cannot lawfully leave such isolation until released by a written discharge from the Board of Health—smallpox, diphtheria, membranous croup and scarlet fever being particularly held in view at this time.

"The Board of Health has appointed fifty physicians, who are known as school inspectors and agents of the Board of Health. Their duties are to visit the public schools daily, to examine all sick or complaining pupils, and to advise the teachers concerning them.

"They will decide all questions concerning the school attendance of such children as live within the same building, but not of the same household, in which there is a case of contagious disease. Such cases must either be so isolated as to not endanger the health and liberty of other families, or must be taken to the hospital.

"They also visit every case of diphtheria, membranous croup and scarlet fever reported; first, concerning the patient's isolation, and second, concerning his discharge from isolation, in accordance with the following regulation of the Board of Health."

Now then, gentlemen, having been far east, let us come home and see "where we are at," and apply what we can of good to ourselves. First, let me speak a word of membranous croup. There is a very large faction among the laity that think membranous croup is not contagious. Now, we all know that it is. The name seems unfortunate, and it seems to me it would be well to do away with membranous croup cards and use diphtheria only. I heard a prominent physician of this city say: "I sometimes report my cases of typhoid, but I often do not. It is not a serious matter." Now, gentlemen, we either have a Board of Health in this particular line of contagious troubles here in this city of which the physicians are ashamed, or should be, or the Board of Health should be decidedly ashamed of the physicians who are supposed to back it up. I am inclined to believe there is fault in both sides. It is the duty of the board to be awake to any contagious cases not reported, and to prosecute the physician willfully not reporting such case to the full extent of the law. The higher that doctor may be in the professional rank the better for the board, for it makes a fine example for the lesser lights. Gentlemen of the board, back up your laws. Now then, as physicians, are we not seriously at fault? Should we not, as members of a profession whose highest aim is to prevent disease, stand squarely behind every proper movement of the board and give it our best support? I fear many of us have not done so, and those of us who will not do so should be compelled by the board itself. The medical inspection of our public schools by physicians, I fear, would be impracticable here in Atlanta, owing to the expense. But an improvement I would suggest and urge, and one that seems to me to be possible, is for the Board of Health to have appointed a competent medical inspector, one who is conversant with scarlet fever and diphtheria particularly, whose duty it shall be to visit each and every case of contagious disease reported, and to see that proper precautions are taken against the spread of such infection, and who also should notify the principal of the school in that district, giving the names of the children in that house, and that these children should be quarantined for the two weeks.

Just here let me say it has been brought to my notice that children from the families where contagion exists have been repeatedly sent to school. An inspector would avoid this serious fault. Again, on the report of the attending physician that a case was ready for discharge the inspector should again visit the house and should see that the patient is without question ready for discharge. Now as to teachers: They should be notified at once through their principal, not by way of the board of education or superintendent of schools. The sooner any teacher knows that a case of contagion exists in her district the better for her other pupils. A teacher, too, should have power, as we have no regular medical inspector, to send home to be examined by the family physician or medical inspector of the board any sick or suspicious cases that may arise in her room. The child should not be allowed to return until it brings a satisfactory note from some reputable physician or the medical inspector. There are many other suggestions that thought and discussion I am sure will bring out.

Thanking you gentlemen for your kind attention, I will not further take up your time, but leave the subject in your hands.

